# Undiagnosed diseases: Needs and opportunities in 20 countries participating in the Undiagnosed Diseases Network International

**DOI:** 10.3389/fpubh.2023.1079601

**Published:** 2023-03-02

**Authors:** Domenica Taruscio, Marco Salvatore, Aimè Lumaka, Claudio Carta, Laura L. Cellai, Gianluca Ferrari, Savino Sciascia, Stephen Groft, Yasemin Alanay, Maleeha Azam, Gareth Baynam, Helene Cederroth, Eva Maria Cutiongco-de la Paz, Vajira Harshadeva Weerabaddana Dissanayake, Roberto Giugliani, Claudia Gonzaga-Jauregui, Dineshani Hettiarachchi, Oleg Kvlividze, Guida Landoure, Prince Makay, Béla Melegh, Ugur Ozbek, Ratna Dua Puri, Vanessa Romero, Vinod Scaria, Saumya S. Jamuar, Vorasuk Shotelersuk, Dario Roccatello, William A. Gahl, Samuel A. Wiafe, Olaf Bodamer, Manuel Posada

**Affiliations:** ^1^National Centre for Rare Diseases, Istituto Superiore di Sanità, Rome, Italy; ^2^Reference Center for Rare and Undiagnosed Diseases, University of Kinshasa, Kinshasa, Democratic Republic of Congo; ^3^Service de Génétique Humaine, University Hospitals of Liège, Liège, Belgium; ^4^Center of Excellence on Nephrologic, Rheumatologic and Rare Diseases (ERK-Net, ERN-Reconnect and RITA-ERN Member) With Nephrology and Dialysis Unit, San Giovanni Bosco Hub Hospital, University of Turin, Turin, Italy; ^5^National Center for Advancing Translational Sciences, National Institutes of Health, Bethesda, MD, United States; ^6^ACURARE-Rare and Undiagnosed Diseases Center, Acibadem University, Istanbul, Turkey; ^7^COMSATS University Islamabad, Islamabad, Pakistan; ^8^Rare Care, Clinical Centre of Expertise for Rare and Undiagnosed Diseases, Perth Children's Hospital, Perth, WA, Australia; ^9^Wilhelm Foundation, Stockholm, Sweden; ^10^Institute of Human Genetics, National Institutes of Health, University of the Philippines Manila, Manila, Philippines; ^11^Department of Anatomy, Genetics and Biomedical Informatics, Faculty of Medicine, University of Colombo, Colombo, Sri Lanka; ^12^House of Rares, Medical Genetics Service, HCPA, Department Genetics UFRGS and DASA, Porto Alegre, Brazil; ^13^International Laboratory for Human Genome Research, Universidad Nacional Autonoma de Mexico, Juriquilla, Queretaro, Mexico; ^14^Georgian Foundation for Genetic and Rare Diseases (GeRaD), School of Medicine, New Vision University, Tbilisi, Georgia; ^15^Faculté de Médecine et d'Odontostomatologie, l'Université des Sciences, des Techniques et des Technologies de Bamako, Bamako, Mali; ^16^Department of Medical Genetics, School of Medicine, University of Pécs, Pécs, Hungary; ^17^Institute of Medical Genetics and Genomics, Sir Ganga Ram Hospital, New Delhi, India; ^18^School of Medicine, Universidad San Francisco de Quito, Quito, Ecuador; ^19^CSIR Institute of Genomics and Integrative Biology, New Delhi, India; ^20^Singhealth Duke-NUS Genomic Medicine Centre, KK Women's and Children's Hospital, Singapore, Singapore; ^21^SingHealth Duke-NUS Institute of Precision Medicine, Singapore, Singapore; ^22^Center of Excellence for Medical Genomics, Department of Pediatrics, Faculty of Medicine, King Chulalongkorn Memorial Hospital and Chulalongkorn University, Bangkok, Thailand; ^23^National Institutes of Health, National Human Genome Research Institute, Bethesda, MD, United States; ^24^Rare Disease Ghana Initiative, Accra, Ghana; ^25^Division of Genetics and Genomics, Harvard Medical School, Boston Children's Hospital, Boston, MA, United States; ^26^Rare Diseases Research Institute (IIER), SpainUDP, Instituto de Salud Carlos III (ISCIII), Madrid, Spain

**Keywords:** Undiagnosed Diseases, rare diseases, developing nations, data sharing, survey

## Abstract

**Introduction:**

Rare diseases (RD) are a health priority worldwide, overall affecting hundreds of millions of people globally. Early and accurate diagnosis is essential to support clinical care but remains challenging in many countries, especially the low- and medium-income ones. Hence, undiagnosed RD (URD) account for a significant portion of the overall RD burden.

**Methods:**

In October 2020, the Developing Nations Working Group of the Undiagnosed Diseases Network International (DNWG-UDNI) launched a survey among its members, belonging to 20 countries across all continents, to map unmet needs and opportunities for patients with URD. The survey was based on questions with open answers and included eight different domains. Conflicting interpretations were resolved in contact with the partners involved.

**Results:**

All members responded to the survey. The results indicated that the scientific and medical centers make substantial efforts to respond to the unmet needs of patients. In most countries, there is a high awareness of RD issues. Scarcity of resources was highlighted as a major problem, leading to reduced availability of diagnostic expertise and research. Serious equity in accessibility to services were highlighted both within and between participating countries. Regulatory problems, including securing informed consent, difficulties in sending DNA to foreign laboratories, protection of intellectual property, and conflicts of interest on the part of service providers, remain issues of concern. Finally, most respondents stressed the need to strengthen international cooperation in terms of data sharing, clinical research, and diagnostic expertise for URD patients in low and medium income countries.

**Discussion:**

The survey highlighted that many countries experienced a discrepancy between the growing expertise and scientific value, the level of awareness and commitment on the part of relevant parties, and funding bodies. Country-tailored public health actions, including general syllabus of medical schools and of the education of other health professionals, are needed to reduce such gaps.

## Introduction

Rare diseases (RD) are a health priority for many countries, altogether affecting up to 6–8% of the population (1). RD, including those of genetic, epigenetic, or environmental origins, are defined as having low prevalence in the population, e.g., not more than 5 persons per 10,000 in the EU population (2), fewer than 200,000 individuals in the US population (330 million) (3), and fewer than 50,000, or one in 2,500, in Japan (4). There are 7,000–8,000 RD; many are complex clinical entities, life-threatening and/or chronically debilitating with multisystem dysfunction. Due to the heterogeneous etiologies and phenotypes, as well as low prevalence of each condition, achieving a timely diagnosis is particularly difficult. Hence, despite increased access to new tools, the diagnosis of RD remains challenging and a strength synergy among all parties involved (clinicians, researchers, patient associations, etc.) and technologies (including genome sequencing and clinical tests, metabolic measurement, neurological measurement etc.) are fundamental. Available information indicates that the mean delay in diagnosis is ~7 years, with high variability (1–18 years) across countries or regions (5, 6). Early and accurate diagnosis is essential to ensure proper access to clinical management of RD. Undiagnosed RDs (URD) account for a significant portion of the overall RD burden in all countries.

Combined national and international efforts are needed to shorten the diagnostic odyssey, improve the management of RD patients, reduce their morbidity and early mortality, and improve their quality of life and socio-economic potential. Those efforts include enhancing diagnostic services through initiatives such as the NIH Undiagnosed Diseases Network (UDN) (7) in the USA, the Deciphering Developmental Disorders project (DDD-Africa) (8) in Africa, the iHOPE Foundation (9), the SWIFT Foundation (10), the Western Australia public health system (11), the Global Commission on Diagnostic Odyssey (12), and private funding. The Solve-RD project (Solving the Unsolved RD) (2018–2022), funded by the EU Commission, is an example of a research project on URD (13). Finally, the International Rare Disease Research Consortium (IRDiRC) is lending support for the global coordination of research initiatives (14).

### The Undiagnosed Diseases Network International

The UDNI was established in 2014 after two international scientific conferences (Rome 2014 and Budapest 2015) (15–17) to provide diagnoses to patients, implement diagnostic tools and foster research on novel diseases and their mechanisms. The Network aims to fill the knowledge gaps that impede diagnosis, particularly for ultra-RD (18), and to foster the translation of research into medical practice, aided by active patient involvement. As outlined in an initial white paper (16), the UDNI works collaboratively and internationally to: (i) provide diagnoses for patients that have eluded diagnosis by clinical experts; (ii) contribute to standards of diagnosis by implementing additional diagnostic tools; (iii) foster research into the etiology and pathogenesis of novel diseases; and (iv) disseminate those research results broadly and rapidly.

The UDNI involves centers with internationally recognized expertise, an international Governing Board, and several interacting Working Groups (WGs) and Committees (15–17). Active patient participation is achieved through the Patient Engagement Group (PEG) comprising 19 patient's organizations (17). The UDNI now involves 41 countries on all continents including Argentina, Australia, Austria, Belgium, Brazil, Bulgaria, Canada, Chile, China, Ecuador, France, Georgia, Germany, Ghana, Hong Kong, Hungary, India, Israel, Italy, Japan, Korea, Kuwait, Mali, Malta, Mexico, New Zealand, Pakistan, Philippines, Saudi Arabia, Serbia, Singapore, South Africa, South Korea, Spain, Sri Lanka, Sweden, Switzerland, Thailand, The Netherlands, Turkey and USA (15).

### The UDNI Developing Nations Working Group

**Figure 1:**
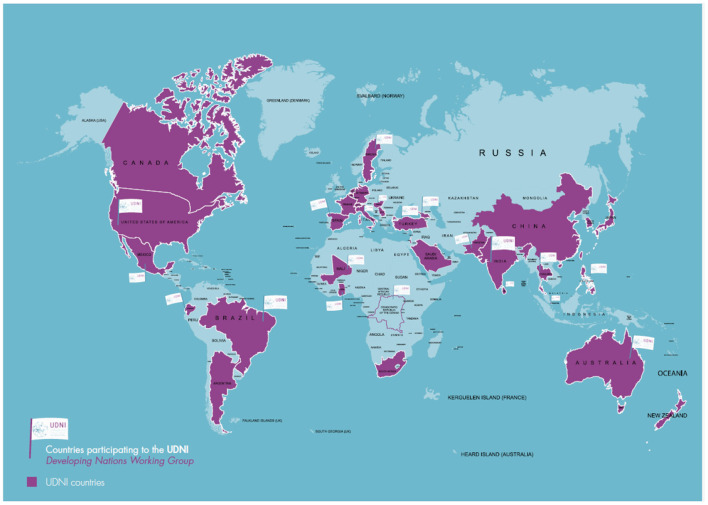
Countries participating to the Undiagnosed Rare Diseases Network International (purple), countries included in the Developing Nations Working Group (flag).

The Developing Nations Working Group (UDNI DN WG, here in after WG) is one of the WG included in UDNI network, composed of representatives of 20 nations, which include developed as well as low and medium income countries (LMIC) (Figure 1) (15). The specific objectives are to: (i) support diagnosis of URD patients in DN; (ii) cooperate with national experts; (iii) build local capacity, including training courses; and (iv) develop global Standard Operation Procedures to achieve correct diagnosis and standards of care. The achievement of these goals is facilitated by the sharing of experiences and competencies with countries that have already started to tackle.

The WG has identified initiatives and institutions to strengthen synergies, such as ICORD (International Collaboration for Rare Diseases and Orphan Drugs), the European Union of Medical Specialists (UEMS) and the Global Genomic Medicine Collaborative (G2MC) (18–22). Moreover, international networking is ensured by the presence of several WG members on IRDiRC Task Forces and Working Groups (23, 24).

The WG provides a comprehensive perspective on RD-related issues at a global level. This paper reports the results of a pilot survey addressed to WG members and launched in October 2020, in order to map unmet needs and opportunities.

## Materials and methods

The survey was developed within the WG to explore the main needs for diagnosing undiagnosed patients, correlating those needs to national health care organizations, tracing resources dedicated to these purposes, estimating the presence or absence of dedicated expertise centers, and evaluating translational research activities. The UDNI members of the 20 countries provided their responses based on their own knowledge of the country situation on a comprehensive array of RD-related challenges. All participant members are engaged in RD at high level in their respective countries.

The survey was organized into eight domains, each interrogated through a dedicated open question with a free text box for the answer. Each country was represented by one respondent and each respondent replied to the questions on the basis of his/her specific knowledge of the country's situation regarding RD and URD. The results were structured based upon the most recurrent descriptions provided by each country's representative and summarized in tables and figures.

*Domain 1. Unmet needs to tackle undiagnosed patients*. This domain explores the principal needs to make diagnoses in patients affected by complex and undiagnosed RD in the specific country.
*Domain 2. Health care organizations*. This domain requires a brief description of the National Health System (NHS) of each country regarding the coverage of complex and RD.
*Domain 3. Insurance systems and regulations*. This domain explores the insurance system(s) involvement in complex and RD diagnosis and the percentage of population covered by each. It requires specifying one or more aspects of the topic, namely, *to list* the insurance system(s) involved in complex and rare disease diagnosis and the percentage of population covered by each (if more than one), and *to specify* which kind of genetic diagnosis is included in and covered by each system.
*Domain 4. Resources*. This domain addresses whether dedicated funds, biobanks, patient registries and facilities are available for RD and URD in the country. It requires listing of specific RD human resources, facilities, funds, expertise, diagnostic tools, patient registries and biobanks.
*Domain 5. Center for expert genomic diagnosis*. This domain estimates the percentage of the population that has access to an expert center for RD diagnosis.
*Domain 6. Regulatory aspects*. This domain investigates the presence of regulations that could limit data sharing and the transport of biological samples out of the country.
*Domain 7. Translational research activities at national and international levels*. This domain evaluates whether biological sample and scientific exchange activities are in place for translational research activity purposes.
*Domain 8. Other important issues, e.g., barriers and bottleneck*. This domain highlights the presence of any limitations in RD and URD that were not addressed by domains 1–7.

Hence, the identification of target unmet needs included the availability of centers for expert genomic diagnosis (domains 1 and 5); the depiction of the countries' health care organizations (domain 2); the presence of insurance systems (domain 3); the availability of resources and the implementation of sample and scientific exchange for translational research (domains 4 and 7); and the regulations governing data sharing (domain 6).

The survey was first piloted among WG four co-chairs and some researchers from their teams in order to test its technical functionality and general consistency. Its contents and aims were then shared and agreed with all 20 countries' WG representatives before being administered.

## Results

**Figure 2:**
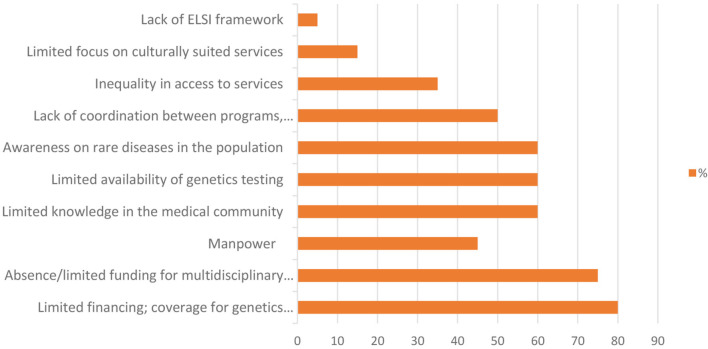
Unmet needs to tackle undiagnosed patients. X axis: percentage of response.

All WG members (*N* = 20; 100%) responded and completed the survey. Table 1 shows the UDNI countries contributing to the survey; it also includes the roles and affiliations of the country representatives and gives information about participation in the UDNI and other undiagnosed disease program initiatives. Table 2 and Figure 2 describe the unmet needs to tackle undiagnosed patients with rare diseases across all participating countries.

**Table 1:** UDNI countries and country representatives contributing to the UDNI Developing Nations Working Group survey (*N* = 20 in alphabetical order per country).

**Country**	**Country representative**	**UDNI member and/or other undiagnosed disease program initiative**	**Organization (URL if available)**
Australia	Gareth Baynam	UDNI member, Global Commission to End the Diagnostic Odyssey for Children with a Rare Disease	Undiagnosed Diseases Program-WA, Genetic Services of WA https://ojrd.biomedcentral.com/articles/10.1186/s13023-017-0619-z
Brazil	Roberto Giugliani	UDNI member	House of Rares and Hospital de Clinicas de Porto Alegre (https://www.hcpa.edu.br/); Casa dos Raros (www.casadosraros.org.br); Hospital de Clinicas de Porto Alegre (www.hcpa.edu.br)
DR Congo	Aimé Lumaka	UDNI member	Center for Human Genetics, University of Kinshasa
Ecuador	Vanessa Romero	UDNI member	Universidad San Francisco de Quito; https://www.usfq.edu.ec/es
Georgia	Oleg Kvlividze	UDNI member	Georgian Foundation for Genetic and Rare Diseases (GeRaD); School of Medicine, New Vision University, https://newvision.ge/eng/
Ghana	Samuel Wiafe	UDNI member	Rare Disease Ghana https://www.rarediseaseghana.org/
Hungary	Bela Melegh	UDNI member and MJC RUD-UEMS	University of Pécs https://international.pte.hu/
India	Ratna Dua Puri	UDNI member	Institute of Medical Genetics and Genomics, Sir Ganga Ram Hospital, New Delhi, India https://sgrh.com/departments/institute_of_medical_genetics_genomics
	Vinod Scaria	UDNI member	CSIR Institute of Genomics and Integrative Biology, Mathura Road, Delhi, India www.csir.res.in/institute-genomics-and-integrative-biology-delhi
Italy	Domenica Taruscio	UDNI member. ICORD President and MJC RUD UEMS member	Istituto Superiore di Sanità (ISS), National Center for Rare Diseases www.iss.it
Mali	Guida Landoure	UDNI member	University of Science, Technique and Technology of Bamako https://sites.sph.harvard.edu/global-health-research-partnership/sites-2/university-of-sciences-techniques-and-technologies-of-bamako-mali/
Mexico	Claudia Gonzaga-Jauregui	UDNI member and G2MC	Universidad Nacional Autónoma de Mexico (UNAM) https://www.unam.mx/
Pakistan	Maleeha Azam	UDNI member	COMSATS University Islamabad, Pakistan https://www.comsats.edu.pk/
Philippines	Eva Maria Cutiongco-de la Paz	UDNI member	Institute of Human Genetics, National Institutes of Health, University of the Philippines Manila https://nih.upm.edu.ph/institute/institute-human-genetics
Singapore	Saumya Shekhar Jamuar	UDNI member	KK Women's and Children's Hospital https://www.kkh.com.sg
Spain	Manuel Posada	UDNI member, SpainUDP & ICORD member	Institute of Rare Diseases Research, ISCIII https://eng.isciii.es/eng.isciii.es/QuienesSomos/CentrosPropios/IIER/Paginas/default.html
Sri Lanka	Dineshani Hettiarachchi	UDNI member, G2MC &	Human Genetics Unit, Faculty of Medicine University of Colombo https://med.cmb.ac.lk/hgu/
	Vajira HW Dissanayake	Rare Undiagnosed Diseases Flagship Project	
Sweden	Helene Cederroth	UDNI member	Wilhelm Foundation https://wilhelmfoundation.org/
Thailand	Vorasuk Shotelersuk	UDNI member	Chulalongkorn University https://www.chula.ac.th/en/
Turkey	Ugur Özbek	UDNI member	Acibadem University ACURARE Center for Rare and Undiagnosed Research https://www.acibadem.edu.tr/en/rare
	Yasemin Alanay		
USA	Stephen Groft	UDNI member, ICORD-ERCAL	NCATS, NIH https://ncats.nih.gov/
	William A. Gahl	UDNI member, UDP-USA Director	NIH https://www.nih.gov/

**Table 2:** UDNI DN survey: Unmet needs to address undiagnosed rare disease cases.

Country name^*^	**AT**	**BR**	**CD**	**EC**	**GS**	**GH**	**HU**	**IN**	**IT**	**ML**	**MX**	**PK**	**PH**	**SG**	**SP**	**LK**	**SE**	**TH**	**TR**	**USA**
Awareness in the population																				
Limited knowledge in the medical community																				
Limited availability of genetics testing																				
Limited financing/ coverage for genetics tests and services																				
Absence or limited funding for multidisciplinary investigations or care for patients																				
Lack of coordination between programs, institutions involved in clinical care																				
Inequality in access to services																				
Limited focus on culturally suited services																				
Manpower																				
Lack of ELSI framework												-								

* Solid square represents a positive response.

** Country situation is referred at 2021 year.

AT, Austria; BR, Brazil; CD, Democratic Republic of Congo; EC, Ecuador; GS, Georgia; GH, Ghana; HU, Hungary; IN, India; IT, Italy; ML, Mali; MX, Mexico; PK, Pakistan; PH, Philippines; SG, Singapore; SP, Spain; LK, Sri Lanka; SE, Sweden; TH, Thailand; TR, Turkey; USA, United States of America.

Eighty percent of all countries (*N* = 16) indicated limited financing and/or insurance coverage for genetic tests/services (Figure 2) which underscores that most countries are aware of ongoing challenges regarding rare diseases among clinicians and patient communities (e.g., Democratic Republic of Congo), complexity of rare diseases preventing identification and subsequent diagnosis (e.g., Ghana, Mexico), limited availability of specific tests in the public healthcare system, and lack of governance to assess the burden of genetic disorders (e.g., India).

Additionally, inequality in accessing services has been reported within almost all countries in terms of: financial accessibility of genetic testing (e.g., Georgia); limited number of local labs proficient in Next Generation Sequencing (e.g., Singapore); patients living in remote areas (e.g., Brazil); absence of a unique health care plan for URD across the country (e.g., Sweden); individual efforts rather than an institutional stand (e.g., Turkey); high cost of the investigations (e.g., Ghana); and absence of appropriate diagnostic facilities in the country (e.g., Pakistan).

A key difference among countries seems to be the percentage of the population that lacks appropriate access to diagnostic services; see “Limited availability of genetics testing” (Table 2) (25, 26). Beyond the specific RD aspects, this may also reflect the general development differences embodied by allocated and sustainable funding and the capacity of the countries to develop regulations and policies targeted to the unmet diagnostic needs.

### Health care organization

**Figure 3:**
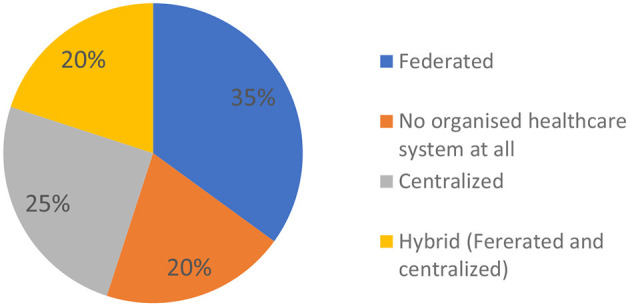
Health care organization for diagnosis of complex and rare diseases. Federated health care organization: Brazil, Georgia, Hungary, Italy, Singapore, Spain, Sweden. No organized healthcare system at all: DR Congo, Ecuador, Mexico, Pakistan. Centralized health care organization: Ghana, Mali, Philippines, Sri Lanka, Thailand. Hybrid (Federated and Centralized) health care organization: Australia, India, Turkey, USA.

The organization of health care varies considerably across countries (Figure 3). For the diagnosis of complex and RD, health care organization was described as Federated (35% of cases), Centralized (25% of cases), or Hybrid (20% of cases); 20% of countries declare no organized healthcare system at all. In countries whose NHSs are federated (namely, Brazil, Georgia, Hungary, Italy, Singapore, Spain, Sweden), it appeared difficult to find a centralized organization in charge of a nationally coordinated UDP. In countries with a centralized NHS (namely, Ghana, Mali, Philippines, Sri Lanka, Thailand), there are several limitations due to the geographic size of the country, incomplete or inequitable access to services, and scarcity of resources. Most countries do not systematically cover Whole Exome Sequencing/Whole Genome Sequencing (WES/WGS) analyses for undiagnosed cases; only those having a UDP can obviate this deficiency.

**Figure 4:**
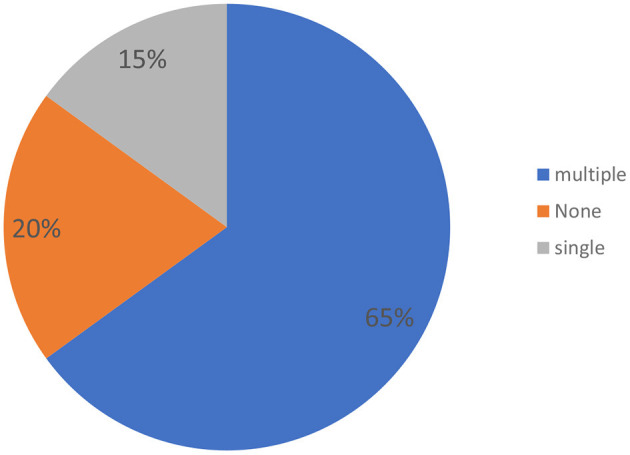
Health care organization: Expert Centers for RD diagnosis.

Most countries (17/20, 85%) report the existence of expert centers (Figure 4), but some of these may not fulfill the needs of all RD patients requiring a diagnosis. In developing nations, a portion of the population cannot access a RD diagnosis due to the lack of funds and specialized resources and/or limits in the organization/coordination among the existing centers. Most countries (65%) affirm that multiple Expert Centers for RD diagnosis are available; in 15% of cases there is one Expert Center, and in 20% of cases none.

### Insurance systems and regulations

A country's NHS does not always cover all undiagnosed patient expenditures, nor does it extend to 100% of the population (Table 3). These factors limit the equity of access to a genetic diagnosis. In some countries, the private sector exerts substantial influence.

**Table 3:** Major results from UDNI DN survey: Insurance systems.

**Country Name**	**AT**	**BR**	**CD**	**EC**	**GS**	**GH**	**HU**	**IN**	**IT**	**ML**	**MX**	**PK**	**PH**	**SG**	**SP**	**LK**	**SE**	**TH**	**TR**	**USA**
Beveridge model^*^		Yes		Yes				NA	Yes			Not applicable	No		Yes	Yes	Yes	Yes	Yes	
Bismarck model^**^	Yes	No						Yes	No	Yes		No	No					No	Yes	Yes
NH Insurance model^***^		No			Yes		Yes	No	No			No	Yes					No		
Out-of-pocket payments		Yes	Yes			Yes		Yes	yes		Yes	Yes	Yes	Yes				Yes		
Other		§		§	§			§	§			§	§§	§				§§§	§ç	Çç

^*^ Government provides health care for all its citizens through income tax payments.

^**^ People pay a fee to a fund that in turn pays health care activities, that can be provided by State-owned institutions, other Government body-owned institutions, or a private institution.

^***^ System has elements of both Beveridge and Bismarck models.

§ Additional subscription to private insurance company is possible.

§§ Donations.

§§§ During 5 years (2020–2024), WGS costs will be covered by the Genomics Thailand Project.

§ç Additional subscription to private insurance company for childhood and adolescence started genetic hereditary diseases.

çç There may be co-pays for services required until annual limit is met and then services may be covered completely.

AT, Austria; BR, Brazil; CD, Democratic Republic of Congo; EC, Ecuador; GS, Georgia; GH, Ghana; HU, Hungary; IN, India; IT, Italy; ML, Mali; MX, Mexico; PK, Pakistan; PH, Philippines; SG, Singapore; SP, Spain; LK, Sri Lanka; SE, Sweden; TH, Thailand; TR, Turkey; USA, United States of America.

Empty cell means “Unknown.”

### Resources

Many respondents describe the existence of several centers in their countries, ranging from clinical centers to centers of excellence based on private and public initiatives (Table 4). However, it is unclear whether these centers are able to cover all needs. Registries and biobanks are other resources reported by many countries, even if the capacity and actual possibility of benefiting from the use of these resources are not clear. Most countries (particularly Mali, Ghana, Ecuador, Georgia and Singapore) report that they do not have enough human resources; specifically, they have very few geneticists per million inhabitants.

**Table 4:** Types of the main needs in terms of resources and availability of translational research: Numbers by country.

**Resources for rare diseases**	**Sufficient *n* (%)**	**Insufficient ** ***n* (%)**	**Not available/Not response *n* (%)**
Human resources	1 (5)	19 (95)	0 (0)
Facilities	2 (10)	18 (90)	0 (0)
Public funding	0 (0)	16 (80)	4 (20) / 0 (0)
Private funding	0 (0)	12 (60)	0 (0) / 8 (40)
	**Available** ***n*** **(%)**	**Not available** ***n*** **(%)**	**No response** ***n*** **(%)**
Biobank	12 (60)	6 (30)	2 (10)
State/national registry	6 (30)	8 (40)	6 (30)
Institutional registry	13 (65)	2 (10)	5 (25)
**Availability of translational research**	**Available** ***n*** **(%)**	**Not available** ***n*** **(%)**	**No response** ***n*** **(%)**
National	15 (75)	5 (25)	0 (0)
International	16 (80)	3 (15)	1 (5)

### Regulatory aspects for data sharing

**Figure 5:**
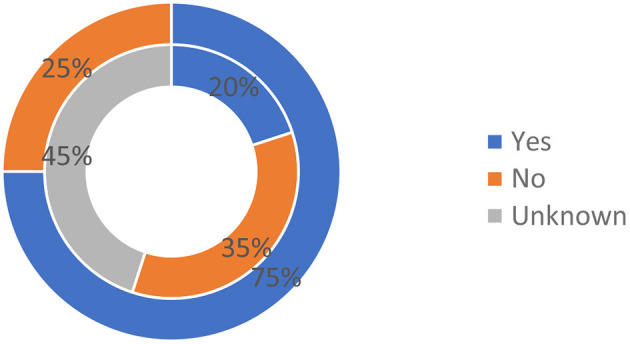
Existence of regulations for data sharing with international. Outer circle: Regulations governing data sharing from the general population with international partners; Inner circle: Regulation governing data sharing from the indigenous populations.

This area seems to be well-covered, with 15/20 (75%) countries having regulations for data sharing with international partners (Figure 5). Interestingly, a regulation for data sharing with international partners specific to indigenous populations is reported in three countries, i.e., Ecuador, India, Philippines (Figure 5 inner circle). For up to 80% of the surveyed groups, data sharing on RD is covered by the standard clinical practice mandates of written informed consent, maintaining confidentiality and ethical committee approval. The use of informed consent as well as the existence of Institutional Review Boards (IRBs) are the two main procedures to assure the ethical use of samples and data for health care and research activities. Some of the countries have strict regulations and DNA cannot be sent to external laboratories without authorization: this has emerged as a critical point. Time to obtain an authorization is also an issue, since some authorizations can take up to 2 years. Finally, many countries are interested in translational research and sharing information. However, they encounter difficulties and limitations. Many countries report that there is little or limited international cooperation.

### Other issues

Additional highlighted issues include: (i) lack of funding and of specialized human resources; (ii) need for coordination with other groups and for the development of standardized guidelines; (iii) protection for discoveries and the possible conflict of interest among researchers; (iv) limited awareness of RD; and (v) lack of data sharing.

## Discussion

Our study involved 20 countries in six continents, with highly diverse socio-economic and demographic scenarios. To our best knowledge, this is the first international overview on a comprehensive array of issues related to the needs and opportunities for undiagnosed RD.

Our results show that while the scientific and medical centers of most respondent countries are making substantial efforts to meet the unmet needs of patients, most health policies and frameworks do not adequately address RD needs, nor have they translated to adequate funding. Indeed, the scarcity of resources for genetic tests/services for multidisciplinary investigations and care for RD patients have been highlighted as the main problems. This is reflected by comments on the lack of a governmental strategy for undiagnosed patients and a general lack of awareness about the difficulties of achieving a diagnosis for RD patients. These systemic weaknesses translate into problems such as the scarcity of experts in genomic analysis and lack of interest of researchers and clinicians driving most initiatives.

In a substantial minority of countries, the medical structures appear utterly insufficient. Health care organization for diagnosis of RD is absent in 20% of cases. In 35% of cases, either one or no Expert Center for RD diagnosis is available for millions of inhabitants.

Furthermore, each health service, whether federated, centralized, or hybrid, has its own specific challenges. Federated systems, representing a relative majority, have difficulty achieving a nationally coordinated UDP, while centralized health services have limitations due to incomplete or inequitable access to services and scarcity of resources. Importantly, most countries do not have systemic or systematic coverage of WES/WGS analyses for undiagnosed cases.

Costs are a further barrier, creating substantial issues of inequity and lack of accessibility. NHSs do not always cover all undiagnosed patient expenditures, nor do they extend to 100% of the population. The contribution of the private sector, while useful to provide other options, does not address the equity problems. Overall, the cost of analyses for RD diagnosis is still high in most countries; in some countries, RD diagnosis is accessed only through research studies. Besides diagnostic services, patient registries and biobanks are available in many countries, but their access and functionality remain to be determined. These observations were confirmed when analyzing the responses of the participants addressing the unmet needs of RD and URD; “funding,” “facilities,” and “biobank” were the most frequently used terms.

On the other hand, the regulatory aspects of data sharing present several favorable aspects. Regulations are in place regarding the use of informed consent, as well as the existence of IRBs to assure the appropriate use of human samples and data. However, in some countries, strict regulations on sending DNA samples to foreign laboratories create additional diagnostic delays. Other possible challenges involve the protection of intellectual property from discoveries and the presence of possible conflicts of interest among researchers.

This survey highlighted some evident differences in responses by participants, likely related to different healthcare systems, policies and available funding to access exome / genome sequencing.

There is also a difference in availability of adequate genomic and human resource expertise for rare, undiagnosed diseases for specific populations. More pressing social and healthcare needs for commoner diseases like infections may result in diversion of funds and efforts in countries with lower socioeconomic status. In addition, these countries may not report inequity as there is lack of awareness of URD concept and thereby a limited felt need by them.

The findings of our survey are consistent, from a different perspective, with the insights collected by parents of children with RD in West Australia (26). Besides the needs for health and social care, the parents emphasized their diagnostic odysseys and the key role of a timely and appropriate diagnosis also as a “starting point” for making sense of their conditions.

Our results also provide information on research and professional training. Research is relatively well-advanced compared to other health policy aspects, since most countries have centers developing translational research programs and there is widespread interest in translating results and sharing information (27–35). Nevertheless, international cooperation needs to be promoted and strengthened. The need for international cooperation and sharing of best practices extends also to professional training, as acknowledged and enabled by the UEMS. Beyond expert specialist training, RD should become part of the general syllabus of medical schools and of the education of other health professionals.

The results of this survey, although retrospective and involving a relatively small number of responders, represent a snapshot of the concerns of a broad range of clinicians and scientists with extensive expertise in rare diseases, identifying a range of issues that call for tailored investigation in specific world areas. Hence, the findings provide a framework for the future advancement of rare disease research and healthcare, particularly in low and medium income countries. A possible drawback of this work is that the individuals who created the questions also completed the survey. We note, however, that the survey responses represent answers to those questions and those answers are based upon experience and facts. Moreover, the authors of the survey are not a homogeneous group but have varied backgrounds and experiences, so that one member's questions address issues not previously considered by other individuals in the group. At times, individual survey results might also confirm issues raised by other group members. Therefore, the survey results portray a valuable perspective on a comprehensive array of issues relevant to the unmet needs of persons with RD.

## Data availability statement

The raw data supporting the conclusions of this article will be made available by the authors, without undue reservation.

## Ethics statement

Ethical review and approval was not required for the study on human participants in accordance with the local legislation and institutional requirements. Written informed consent was not required to participate in this study in accordance with the local legislation and institutional requirements.

## Author contributions

DT, MS, CC, LC, SS, and AL analyzed the data and were the major contributor in drafting the manuscript. GF, SG, YA, MA, GB, HC, EC-d, VD, RG, CG-J, DH, OK, GL, PM, BM, UO, RP, VR, VSc, SJ, VSh, DR, WG, SW, OB, and MP carefully revised draft, tables and figures, and approved final manuscript. All authors contributed to the article and approved the submitted version.
